# Approaches to prevention of gynecological malignancies

**DOI:** 10.1186/s12905-024-03100-4

**Published:** 2024-04-23

**Authors:** Federico Ferrari, Andrea Giannini

**Affiliations:** 1 Department of Clinical and Experimental Sciences, University of Brescia, Brescia, Italy; 2 Unit of Gynecology, Department of Surgical and Medical Sciences and Translational Medicine, Sant’Andrea Hospital, Sapienza University of Rome, Rome, Italy

## Abstract

Gynecological malignancies represent one of the prevalent diseases in the female sex and prevention is essential to limit their incidence and mortality. Nowadays, not all malignancies benefit from adequate screening methods for this reason new biomarkers and methods are being developed to undertake timely and effective therapies.

Gynecologic cancers represent one of the most prevalent neoplastic diseases in women and are a major cause of cancer-related mortality in women in nations with high socioeconomic development. The most frequent gynecologic malignancies include endometrial carcinoma (EC), cervix carcinoma (CC), and ovarian carcinoma (CO). Globally, there are more than 600,000 new cases of CC, accompanied by more than 300,000 deaths; for EC, there are more than 400,000 new cases with a mortality of around 100,000 deaths; for CO, the incidence exceeds 300,000 new cases with more than 200,000 deaths [1]. Prevention of gynecologic malignancies is a central aspect of reducing the risk of developing cancers in the female reproductive system. Unfortunately, the spread of cancerous disease cannot always be prevented, given the poor preventive methods for some malignancies.

Currently, there is a lack of screening approaches with proven efficacy for EC detection. In most clinical scenarios, the diagnosis of the disease becomes concrete only after the manifestation of explicit symptoms. Any patient reporting abnormal bleeding undergoes a gynecologic evaluation, followed by a standard work-up procedure aimed at investigating and determining the origin of the bleeding. This diagnostic work-up may include tests such as transvaginal ultrasound, pelvic ultrasound, and endometrial biopsy, with or without the assistance of hysteroscopy [2, 3]. Maintaining a low body mass index (BMI) helps reduce the risk of EC [4]. Research conducted by Schouten et al., through a cohort study, found that women with significantly lower BMI in older age than their value recorded at age 20 had a 50% reduced risk of developing EC compared with those who maintained a constant BMI over the same time frame [5]. Moreover, Trentham et al., through a case-control investigation, identified that women with obesity who had a weight reduction during a period of at least five years had a 25% reduced risk of developing EC compared with individuals whose weight had remained constant [6]. Prolonged use of oral contraceptives has been associated with a reduction in the incidence rate of EC and some women benefit from this persistent reduction in risk for many years after cessation of oral contraceptive therapy.

Compared with other gynecologic malignancies, robust evidence for prevention and screening is currently available for cervical cancer. First, abstaining from smoking and using barrier devices can limit the acquisition of human papillomavirus (HPV) infection. CC prevention is no longer “one size fits all” with annual examinations for all women. Primary prevention begins in adolescence with vaccination to prevent infection in the future. Wide evidence supports the efficacy of HPV vaccination in early adolescence for the prevention of HPV infection, the development of precancerous lesions and CC. If vaccines currently on the market confer long-term immunity, CC rates could be reduced by 85% for those who were vaccinated before the exposure to oncogenic HPV [7].

In addition, screening modalities, such as cervical cytology (Pap test), primary screening with HPV test, and co-testing with HPV and cytology, allow the early identification of cervical lesions by reducing the incidence and mortality of CC, directing patients to appropriate management strategies. Finally, tertiary prevention focuses on the treatment of previously identified lesions through surgery and other therapeutic strategies [8].

Genetic susceptibility constitutes the preponderant risk identified for CO predisposition; therefore, optimizing the identification of women with genetic mutations associated with an increased CO risk is of primary importance for preventive strategies. Over the past few years, there have been significant advances in CO screening and prevention. The largest CO screening study to date, the UK Collaborative Trial of Ovarian Cancer Screening, reported a stage change with annual multimodal screening using the cancer antigen 125 (CA 125) longitudinal CO risk algorithm, but not with annual screening with transvaginal ultrasound. No definite reduction in mortality was found with either screening strategy compared with no screening [9]. The search for new markers to optimize CO screening has led to the use of a combination of biomarkers, achieving sensitivity and specificity of more than 90%. Despite this, the evaluation of their effectiveness in prevention is not yet fully understood, although some investigations suggest that, at present, the synergy between CA125 and human epididymis protein 4 (HE4) constitutes the most effective biological diagnostic tool for ovarian cancer detection [10].

Prevention of gynecologic malignancies requires an integrated approach based on the synergistic combination of screening strategies, vaccination programs, adoption of a healthy lifestyle, and active management of risk factors. Prevention aims to prevent the occurrence or, alternatively, to detect any malignancy early to undertake timely and effective therapies. Although the fear associated with cancer is predominantly related to the prospect of premature mortality, the focus on prevention and early detection also aims to reduce related discomfort and disability. Such an approach emerges as a priority in women’s health management. A deeper understanding of the risk factors related to these cancers, together with current preventive strategies, is essential to increase awareness, thereby helping to reduce incidence, improve survival rates, and reduce mortality rates in the general population, especially among high-risk patients.

## Data Availability

No datasets were generated or analysed during the current study.

## Abbreviations

EC: Endometrial carcinoma
CC: Cervical carcinoma
CO: Ovarian carcinoma
BMI: Body mass index
HPV: human papillomavirus
CA 125: The cancer antigen 125
HE4: Human epididymis protein 4
